# Antibody response in silver catfish (*Rhamdia quelen*) immunized with a model antigen associated with different adjuvants

**DOI:** 10.1590/1414-431X20165281

**Published:** 2016-07-25

**Authors:** T.R. Pavan, J. Di Domenico, K.S. Kirsten, C.O. Nied, R. Frandoloso, L.C. Kreutz

**Affiliations:** Programa de Pós-Graduação em Bioexperimentação, Universidade de Passo Fundo, Passo Fundo, RS, Brasil

**Keywords:** Vaccination, Fish, Immune response, CpGs ODN, β-glucan

## Abstract

Adjuvants are essential to boost the immune response to inoculated antigen and play a central role in vaccine development. In this study, we investigated the efficacy of several adjuvants in the production of anti-bovine serum albumin (BSA) antibodies in silver catfish. Two hundred and seventy juvenile silver catfish (60–80 g) of both sexes were intraperitoneally vaccinated with BSA (200 µg/fish) alone or mixed to the following adjuvants: Freund’s complete adjuvant (FCA), Freund’s incomplete adjuvant (FIA), aluminum hydroxide (AlOH), Montanide, four types of cytosine-phosphate-guanine (CpG) oligodeoxynucleotides (ODNs) and three concentrations of β-glucan, and the immune enhancing property was evaluated by measuring anti-BSA antibodies in blood samples at biweekly intervals. Our results demonstrated that CpGs ODNs and β-glucan were as effective as classical adjuvants (FCA, FIA, AlOH and Montanide) in promoting anti-BSA antibodies and that the kinetics of antibody production induced by all adjuvants used in our study had a similar trend to that observed in other fish species, with a peak at 28 days post-vaccination. These results may be useful for the selection of adjuvants for vaccine formulation intended for silver catfish and for the development of vaccine and vaccination strategies to other fish species.

## Introduction

Aquaculture is the fastest growing animal protein production industry in the world and, as such, is facing enormous challenges. The occurrence of infectious diseases, for instance, accounts for production losses of up to twenty per cent each year (1). The development of oil-based cost-effective vaccine and vaccination strategies was crucial to control infectious diseases in salmonid aquaculture (2) and since then these strategies have been extended to other fish species. The classical water in oil (w/o) adjuvant, known as Freund’s incomplete adjuvant (FIA), has long been used for immunization studies in mammals and fish (3), and it is capable of inducing a robust humoral immune response that stands as reference when other classes of adjuvant are evaluated. In fish, it is widely acknowledged, however, that intraperitoneal injection of oil-based adjuvanted vaccine might cause organ adherence and a poor Th1 lymphocyte response that limits its use against intracellular pathogens (4). An improved form of w/o adjuvant, the Freund’s complete adjuvant (FCA), contains inactivated dried *Mycobacterium tuberculosis* which contributes to a Th1 lymphocytes-biased immune response (2) but its clinical use is prohibited. Thus, research on novel immunoadjuvants for aquaculture species are in great demand.

Although the requirement of an adjuvant for an effective and robust immune response to an inoculated antigen is long known, the mechanisms underlying this phenomenon have been poorly investigated. For instance, a possible *modus operandi* of aluminum adjuvants, the oldest and most widely used adjuvant in human and animals, was only recently described (5). Currently, the effectiveness of an adjuvant is determined not only by its ability to retain the antigen at the inoculation site but mainly by its interaction with specific receptors, named Patter Recognition Receptors (PRRs), on the surface of antigen presenting cells (APCs) (4). Stimulated APCs and their secreted cytokines play a central role in both B- and T-lymphocyte activation leading to humoral and cell-mediated immunity. In this scenario, pathogen-associated molecular patterns (PAMPs) molecules, which interact with several classes of PRRs, represent a novel group of molecules (4,6) that might be explored as prophylactic and therapeutic drugs in fish and mammals. β-glucan, a complex carbohydrate derived from the yeast cell wall, and synthetic oligodeoxynucleotides (ODNs) containing unmethylated cytosine-phosphate-guanine (CpG) dinucleotides, which mimic bacterial DNA, constitute well known groups of PAMPs (6). On mammals, β-glucan interacts mainly with Dectin-1, a C-type lectin-like group of PRRs expressed on the surface of neutrophils, macrophages and dendritic cells (7). In fish, when added to the diet, β-glucan stimulates innate immune-associated proteins such as serum lysozyme and complement system, and serum bactericidal activity; and when injected by the intraperitoneal route, it improves antibody production and protection to challenging bacterial pathogens (8). CpG ODNs interact with an intracellular endosomal PRRs named Toll-like receptor 9 (TLR-9) (9). The adjuvant efficacy of CpG ODNs, however, depends on the sequence of the CpG motifs in the ODN and on the animal species in which they are evaluated. Some CpGs ODNs have already been evaluated in a few fish species and their effect was observed in innate and adaptive immune function (9). However, it is largely unclear whether the immune potentiating effect of CpGs ODNs and β-glucans are species-specific; thus, their effects should be evaluated in each economically important fish species to assure that they can be widely used.

With this in mind, and knowing that there are no studies on the efficacy of vaccine and adjuvant in silver catfish (*Rhamdia quelen*), we aimed to evaluate the effectiveness of oil-based adjuvants (FCA, FIA and Montanide), AlOH and two classes of PAMPs (β-glucan and four different types of CpG ODNs) in potentiating the antibody production to a model antigen in this important South American fish species.

## Material and Methods

### Fish

Two hundred and seventy healthy juvenile silver catfish (60–80 g) of both sexes were acclimatized in self-cleaning tanks containing 1000 L of continuously running water (18 fish/tank) and used throughout this study. Water parameters were measured during the experiment and were found within the expected range as previously reported (10). Fish were fed twice daily with commercial fish pellets (42% crude protein, Supra, Brazil). The experiments were approved by the Ethical and Animal Welfare Committee of Universidade de Passo Fundo (protocol #011/2012).

### Antigen, adjuvants and fish inoculation

BSA (Sigma, USA) was dissolved in sterile water (10 mg/mL), filtered and kept frozen at -20°C until use. FCA and FIA, AlOH, and β-glucan were purchased from Sigma. Montanide Gel 01 was purchased from Seppic (Brazil). CpGs ODN 1668, 2102, 2133 and 2143, as described by Carrington and Secombes (9), were acquired from InvivoGen (France). In all experiments, each fish was inoculated intraperitoneally once with 200 µg of BSA mixed with the adjuvant, in a final volume of 200 µL. FCA and FIA were mixed with the antigen in a ratio of 1.2:1. Montanide Gel 01, at a final concentration of 20% (v/v) as recommended by the manufacturer, was mixed with BSA diluted in pH 6.0 acetate buffer. AlOH was mixed with BSA at a final concentration of 3% (v/v). CpGs ODNs were mixed to a final concentration of 10 µg/fish and β-glucan was mixed to BSA to a final concentration of 0.02, 0.06 and 0.1%. In the first experiment we evaluated FCA, FIA, AlOH and Montanide. In the second experiment we evaluated the four CpG ODNs and in the third experiment we evaluated the three concentrations of β-glucan and Montanide as positive control. In all three experimental settings, one group was inoculated with BSA mixed to sterile phosphate buffered saline (PBS), pH 7.2, that served as non-adjuvanted control and another group inoculated with PBS only (non-immunized control).

For immunization and blood sampling, all fish were anesthetized using Eugenol (50 mg/L). Blood samples (0.3 mL) were drawn from the caudal vein prior to (day zero), or at 14, 28 and 42 days post-inoculation (*pi*) and allowed to clot for at least 2 h in ice-chilled tubes. Serum samples were collected after centrifugation (8000 *g* for 10 min at 4°C) and stored at –20°C until antibody titering by ELISA.

### Enzyme-linked immunosorbent assay

The ELISA assay was performed as recently described (11). In brief, 96-well ELISA plates were coated overnight (4°C) with BSA (5 µg/well) diluted in pH 9.6 carbonate-bicarbonate buffer, and then blocked with PBS containing 0.05% Tween 20 (PBST) and 3% skim milk (PBST-SK3%, Sigma). Fish serum samples diluted 1:100 in PBST-SK1% were added in duplicates to the wells. After 1 h incubation at 23°C and washing with PBST, rabbit anti-silver catfish IgM antibodies diluted 1:400 in PBST-SK1% was added to the wells. The plates were incubated and washed as described above. Horseradish peroxidase conjugated goat anti-rabbit IgG (Sigma) was added to the plates (diluted 1:20,000 in PBST-SK1%) and incubated 1h at 23°C. After washing, color was developed using O-phenyldiamine (0.067%; Sigma). Plates were read at 492 nm with an Anthos 2010 ELISA plate reader.

### Statistical analysis

The data were evaluated by the Shapiro-Wilk’s test and found to have normal distribution. Differences amongst treatments were analyzed by *t*-test or ANOVA followed by Bonferroni’s multiple comparisons test, and plotted using GraphPad Prism Software v5 (GraphPad Software, Inc., USA). P values of 0.05 or smaller were considered to be significant. Results are reported as means± SE.

## Results and Discussion

**Figure 1:**
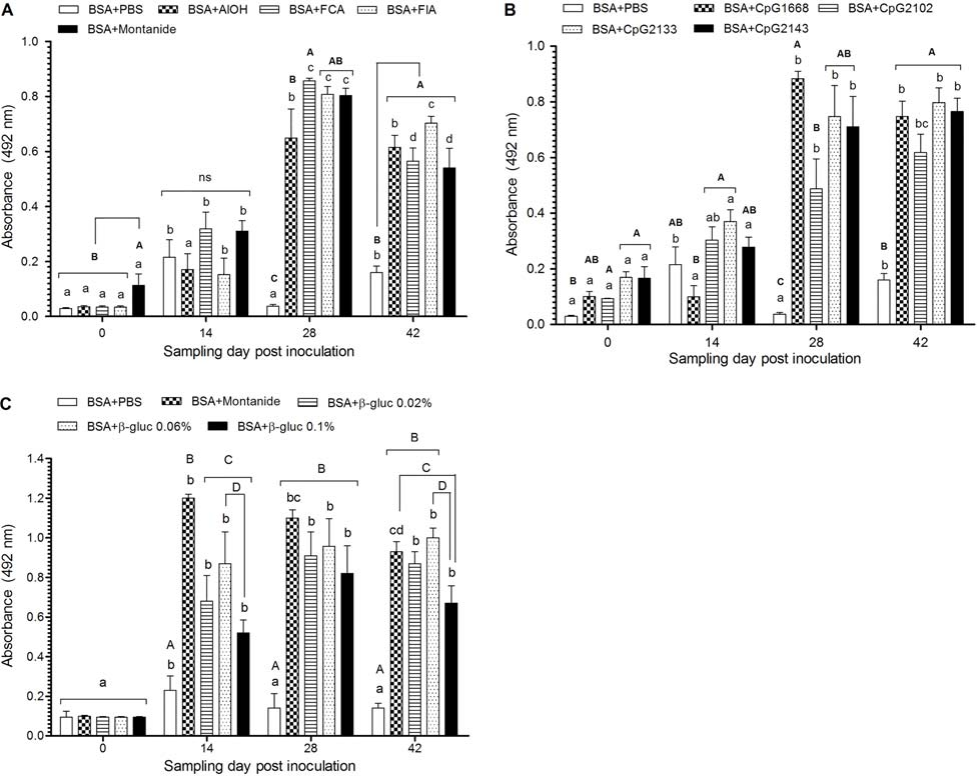
Serum antibody response of fish immunized with bovine serum albumin (BSA) combined with different adjuvants. *A*, Silver catfish were intraperitoneally injected with BSA alone (BSA+PBS, 200 µg/fish) or BSA adjuvanted with aluminum hydroxide (AlOH), Freund’s complete adjuvant (FCA), Freund’s incomplete adjuvant (FIA) and Montanide; *B*, BSA alone or combined with CpG 1668, CpG 2102, CpG 2133 and CpG 2143; *C*, BSA alone or combined with Montanide, and β-glucan (0.02; 0.06 and 0.1%). Serum samples were collected prior to (day 0) or at 14, 28, and 42 days post-inoculation to evaluate anti-BSA antibodies by ELISA. Significant differences (P<0.05) within the same group throughout the experiment are indicated by small letters and significant differences amongst groups at the same sampling day are indicated by capital letters (n=18). Differences amongst treatments were analyzed by ANOVA followed by Bonferroni’s multiple comparisons test.

Adjuvants are central to vaccine development, as they potentiate the immune response to the inoculated antigen and, as a consequence, enhance the possibility of controlling disease outbreaks. Here, we first describe the effectiveness of several types of classical and experimental adjuvants in improving specific antibody production in silver catfish immunized with BSA. In general, we observed that regardless of the adjuvant type, in all fish immunized with adjuvanted BSA, the titer of anti-BSA antibodies increased steadily peaking on day 28, and declined slightly thereafter (Figure 1A, B and C). The exception was observed in one group immunized with BSA+Montanide (Figure 1C) in which the peak of anti-BSA antibodies was detected at 14 days *pi*. Interestingly, in this experiment, we added the BSA+Montanide group as a positive control to evaluate the adjuvanticity potential of β-glucan, as we knew from the previous settings that Montanide potentiated the antibody production at levels similar to FCA. In fish immunized with BSA without adjuvant, a small peak of antibody was observed at 14 days *pi*. In non-immunized fish, the OD reading was similar to background levels (blank reading) indicating absence of anti-BSA antibodies (data not shown).

Because fish vaccines are made up of inactivated pathogens, adjuvant testing has become central to modern aquaculture and, as a consequence, the immunoadjuvanticity effects of different classes of adjuvants have been evaluated in several fish species. In turbots (*Scophthalmus maximus*) immunized with a pentavalent vaccine combined either with FCA, propolis or *Astragalus spp* polysaccharide, the antibody production to each tested antigen and the survival rate after challenging with each tested pathogen, was always superior in the FCA immunized group (12). And, similar to our study, specific antibody titers peaked at 28 days after immunization. In Japanese flounder (*Paralichthys olivaceus*) immunized with a recombinant protein of *Edwardsiella tarda* mixed to FIA, AlOH or aluminum phosphate (13), higher antibody titers were observed in fish vaccinated with the aluminum-adjuvanted antigen, but the survival rate was higher in fish immunized with antigen mixed to FIA. Rainbow trout (*Oncorhynchus mykiss*) immunized with a bivalent vaccine (*Aeromonas hydrophila* and *Lactococcus garvieae*) adjuvanted with Montanide (ISA 763 AVG) had antibody titers and survival rates similar to fish immunized with non-adjuvanted dead bacteria (14). In addition, divalent or multivalent *w/o* vaccine improved antibody titers and survival rate of rainbow trout vaccinated against *Flavobacterium psychrophilum* (15). Again, in those studies, antibody titers peaked at 30 days and decreased slowly thereafter. However, histopathological analysis of vaccinated fish detected intraperitoneal lesions in fish vaccinated with *w/o* emulsionated vaccine (13) and had a negative impact on growth. Nonetheless, despite their harmful potential, these studies indicate the effectiveness of first generation adjuvanted vaccine in enhancing antibody production in fish.

In this scenario, novel and less harmful adjuvants might find their way into the fish vaccine productive chain. Second generation adjuvants used in this study (β-glucan and CpG ODNs) constitute a group of molecules known as PAMPs that by interacting with PRRs improve the overall function of APC and induce the expression of cytokines which, in turn, trigger the development of humoral and cell-mediated immunity (4,9). The immune-enhancing effects of β-glucan and CpG ODNs have been widely evaluated on innate immunity (9) and CpG ODNs have also been evaluated as vaccine adjuvants in mammals (6). However, few studies have investigated whether β-glucans or CpG ODNs could improve antibody production to inoculated antigens in teleost fish. In our study, CpG ODNs were effective in promoting antibody production to inoculated BSA at levels similar to that obtained with classical adjuvants, and the kinetics of antibodies had a similar trend (Figure 1B and C). The anti-BSA antibody production in fish immunized with BSA adjuvanted with β-glucan was equally effective at all the evaluated concentrations, but a wider variation was observed amongst immunized fish. In addition, antibody titers also peaked at 28 days post-immunization. In Atlantic salmon (*Salmo salar*), a combo of CpG ODNs:poly I:C mixed to salmonid alphavirus (SAV) induced a higher titer of anti-SVA neutralizing antibodies and provided full protection to challenge compared to *w/o* formulations (Montanide ISA763A) that induced lower anti-SVA antibodies titers and lower protection to challenge (16). In that study, the superior immune-enhancing properties observed with PAMPs adjuvants could be attributed to the combination of CpG ODNs with poly I:C (a synthetic analog of double-stranded RNA, agonist of TLR-3), as both TLR-9 and TLR-3 could have been triggered which, by acting synergistically, optimized overall cytokine expression. Indeed, both TLR-3 and TLR-9 are known to activate NF-κB and IRFs leading to the expressions of a wide range of inflammatory genes such as tumor necrosis factor, interleukin 1 (IL-1) and IL-6, chemokines (IL-8), costimulatory molecules (CD80 and CD86) and type 1 Interferon (IFN-1), that mediate acute inflammation, create an antiviral state and support the development of acquired immunity (17). Thus, by stimulating APC with CpG ODNs, alone or mixed to other PAMPs, antibody production and resistance to challenge with pathogens might excel. The effectiveness of β-glucan as vaccine adjuvant, in contrast, is controversial. Earlier studies indicated augmented antibody production and increased protection to challenge in Atlantic salmon following vaccination with β-glucan adjuvanted *Aeromonas salmonicida* (18). In contrast, a similar study indicated that β-glucan adjuvanted *A. salmonicida* vaccine had no protective effect on Atlantic salmon challenged with homologous bacteria (19) and had no effect on protecting turbot vaccinated with β-glucan adjuvanted *Streptococcus bacterin* (20), or yellowtail vaccinated with β-glucan adjuvanted *Pasteurella piscicida* (21). However, because whole bacteria were used in those studies, one could argue that the adjuvanticity effect of β-glucan could have been overshadowed by the immunogenicity of the bacteria *per se*, in that it contains several PAMPs, or that the infectious pressure of the challenging bacteria surpassed the protective effect of the adjuvanted vaccine. In our study, in contrast, β-glucan was associated with a highly purified model antigen (BSA) and the enhancing effect on anti-BSA antibody production was evident. We acknowledge, however, that additional investigation is required to refine the usage and recommendation of β-glucan as vaccine adjuvant.

Understanding the requirements to excel antibody secretion in fish species is central to vaccine development and vaccination strategies. Because the nucleotide sequence of immune related genes is not yet available for *Rhamdia quelen*, a thorough analysis of immunological genes triggered by each adjuvant could not be performed at this time. Nonetheless, consistent with our study, FCA, FIA, AlOH and Montanide have been evaluated as vaccine adjuvants and their potential to drive antibody secretion has been recognized in fish (4,12–14), but their harmful potential might hinder future usage (3). CpG ODNs and β-glucan, in contrast, were highly effective in boosting anti-BSA antibodies and should be further explored in combination with different antigens and in different fish species.

In conclusion, we demonstrated the effectiveness of adjuvants in enhancing the antibody production in silver catfish vaccinated with a model antigen. Ongoing studies on the efficacy of adjuvanted vaccines to known silver catfish pathogens and the expression of selective immune-related genes in silver catfish cells should provide a better understanding of this subject in the near future.
